# Nafamostat mesylate versus regional citrate anticoagulation for continuous renal replacement therapy in patients at high risk of bleeding: a retrospective single-center study

**DOI:** 10.1186/s40001-024-01660-7

**Published:** 2024-01-20

**Authors:** Dan Liu, Jian Zhao, Hui Xia, Shi Dong, Songjuan Yan, Yugang Zhuang, Yuanzhuo Chen, Hu Peng

**Affiliations:** Department of Emergency, Shanghai Tenth People’s Hospital, Tongji University School of Medicine, No. 301, Yanchang Middle Road, Jingan District, Shanghai, People’s Republic of China

**Keywords:** Nafamostat mesylate, Regional citrate anticoagulation, Anticoagulation, Continuous renal replacement therapy, High risk of bleeding

## Abstract

**Purpose:**

The choice of continuous renal replacement therapy (CRRT) anticoagulation program for patients at high risk of bleeding has always been a complex problem in clinical practice. Clinical regimens include regional citrate anticoagulation (RCA) and nafamostat mesylate (NM). This study aimed to evaluate the efficacy and safety of these two anticoagulants for CRRT in patients at high risk of bleeding to guide their clinical use better.

**Patients and methods:**

Between January 2021 and December 2022, 307 patients were screened for this study. Forty-six patients were finally enrolled: 22 in the regional citrate anticoagulation group and 24 in the nafamostat mesylate group. We collected patients’ baseline characteristics, laboratory indicators before CRRT, and CRRT-related data. We then performed a statistical analysis of the data from both groups of patients.

**Results:**

In our study, the baseline characteristics did not differ significantly between the two groups; the baseline laboratory indicators before CRRT of patients in the two groups were not significantly different. The duration of CRRT was 600 min in the regional citrate anticoagulation (RCA) group, 615 min in the nafamostat mesylate (NM) group; the success rate was 90.7% in the RCA group, and 85.6% in the NM group, the anticoagulant efficacy between the two groups was comparable. There was no significant difference in the safety of anticoagulation between the two groups. We used Generalized Estimating Equations (GEE) to test whether different anticoagulation methods significantly affected the success rate of CRRT and found no statistical difference between RCA and NM.

**Conclusion:**

Our study suggests that nafamostat mesylate's anticoagulant efficacy and safety are not inferior to regional citrate anticoagulation for continuous renal replacement therapy in patients at high risk of bleeding.

## Introduction

Continuous renal replacement therapy (CRRT) is widely used to manage critically ill patients. In the intensive care unit (ICU), critically ill patients with multiple organ failure (MOF), which is often caused by sepsis, are admitted [1]. Blood purification technology to restore homeostasis and maintain a good internal environment of organs and cells throughout the body helps restore organ function and improve prognosis [2]. The coagulation of cardiopulmonary bypass is a significant problem facing CRRT. Frequent coagulation shortens precious treatment time, increases treatment costs and the workload of medical staff, and causes patients to lose more blood and require more blood transfusions. Systemic anticoagulation with unfractionated heparin (UFH) is the most commonly used form of anticoagulation worldwide. Unfortunately, effective UFH increases the risk of bleeding, and bleeding-related complications are frequently reported in the literature [3, 4].

Many critically ill patients are at high risk of bleeding, such as those with recent active bleeding, recent trauma or surgery, severe thrombocytopenia (PLT < 50), anticoagulant use, and disseminated intravascular coagulation (DIC). CRRT anticoagulation is difficult in these patients.

For patients at risk of bleeding who are not on anticoagulant therapy, regional citrate anticoagulation (RCA) is recommended for CRRT as long as the patient has no contraindications to using citric acid [5]. Anticoagulation is limited to the extracorporeal circuit with RCA, and the patient’s coagulation remains unaffected [6]. Growing evidence shows that RCA may reduce bleeding complications and transfusion requirements compared to heparin while prolonging filter life.

Nafamostat mesylate (NM) is a serine protease inhibitor with a molecular weight of 539 Da. It is independent of antithrombin, has inhibitory effects on coagulation factors IIa, Xa, XIIa, kallikrein, and hemolytic enzymes, and can inhibit complement and platelet activation. NM in the blood is rapidly degraded by hepatic carboxylesterase and removed by dialysis/filtration. The half-life in the blood is only 8 min, so it is best used as an anticoagulant that only plays an anticoagulant role in cardiopulmonary bypass [7]. In critically ill, bleeding, and post-operative patients, NM was the anticoagulant of choice, and the incidence of hemorrhagic complications was significantly reduced from 64% with UFH to 4% [8].

Although nafamostat mesylate and regional citrate anticoagulation are both recommended for CRRT in patients at high risk of bleeding, no studies have compared them. This study aimed to evaluate the efficacy and safety of these two anticoagulants for CRRT in patients at high risk of bleeding to guide clinical use better.

## Materials and methods

### Study design and data collection

We collected data on baseline characteristics, comorbidities, prothrombin time, activated partial thromboplastin time (APTT), D-dimer, fibrinogen level, platelet count, and data related to CRRT. Patients were then divided into the NM and RCA groups according to the different anticoagulation methods.

### Patient enrolment in the ICU setting

This was a retrospective, exploratory study based on a review of the medical records of adult ICU patients in a highly complex public tertiary hospital (Shanghai Tenth People’s Hospital, Tongji University School of Medicine, Shanghai, China). The ICU has a capacity of 23 ICU beds. All consecutive patients admitted to our ICU between January 2021 and December 2022 who received CRRT were included in the study.

### CRRT initiation

Our agency proposal does not make strict recommendations for CRRT activation. All treatments are evaluated on a case-by-case basis by the treating physician.

### CRRT delivery

All therapies were delivered using Prisma Flex CRRT generators and ST100 filters. Therapies were standardized according to our unit protocol. The filtration dialysis replacement solution is commercially available Hemofiltration Basic Solution 4000 ml (Qingshanlikang). This product does not contain potassium ions, which is conducive to removing excess potassium ions in the body and maintaining average blood potassium concentration. Still, when clinical treatment is necessary, potassium salt should be added according to the patient's blood electrolyte analysis results. Add a 10% potassium chloride injection of 1 ml to each bag (4000 ml) of this product, and the potassium ion concentration will increase by 0.335 mmol/l. After adding potassium salt, this product is used as liquid A and combined with sodium bicarbonate injection (liquid B) for continuous blood purification. Under normal circumstances, each bag of this product (4000 ml) with 5% sodium bicarbonate injection 250 ml, and through the blood purification device into the body, the dosage according to the continuous blood purification time, generally every 3L ~ 4L/hour. When this product is used in combination with 5% sodium bicarbonate injection 250 ml per 4000 ml, the concentration of each component is as follows: 10 mmol/l glucose, 110 mmol/l chloride, 0.75 mmol/l magnesium, 150 mmol/l calcium, 141 mmol/l sodium, 35 mmol/l carbonate.

### CRRT-NM

Before starting cardiopulmonary bypass, dissolve 20 mg nafamostat mesylate in 500 ml normal saline, flush with the dissolved solution, and fill the hemodialysis circuit. After the start of blood circulation, nafamostat mesylate is dissolved in 5% glucose injection at a dose of 20–50 mg per hour through an anticoagulant infusion line continuously, with moderate increases or decreases depending on symptoms, to maintain APTT at 1.2–1.5 times the baseline value or 45–60 s. The normal APTT reference range is 31.5 ± 10 s.

### CRRT-RCA

The basic principle of RCA is to infuse citrate into the extracorporeal circuit. One molecule of citrate chelates one molecule of ionized calcium (iCa), thereby reducing the level of iCa in the extracorporeal circuit. A concentration of approximately four mmol of citrate per liter of human blood lowers the level of iCa to the target range of 0.25–0.4 mmol/l. The plasmatic coagulation cascade is inhibited at such concentrations, and prolonged filter life is observed [9]. The post-filter calcium ion concentration reflects the sufficiency of anticoagulation, and citrate and blood flow should be comprehensively adjusted to make the post-filter calcium ion concentration between 0.25 and 0.40 mmol/l [10, 11]. The peripheral blood calcium ion concentration reflects the safety of anticoagulation and is used to assess the risk of hypocalcemia and citrate accumulation. In 2012, Kidney Disease: Improving Global Outcomes (KDIGO) recommended maintaining the peripheral blood calcium ion concentration between 1.1 and 1.3 mmol/L [5].

### High risk of bleeding

Patients at high risk of bleeding: recent active bleeding, recent trauma or surgery, severe thrombocytopenia (PLT < 50), taking anticoagulants, DIC.

### Statistical analyses

The data analysis was based on two distinct datasets. The admission baseline dataset comprised information collected when patients were admitted, involving 46 individuals. The second, the treatment dataset, consisted of measurements taken before each continuous renal replacement therapy (CRRT) session, amounting to 172 instances of treatment application. The corresponding table legend indicates the specific dataset used for each analysis.

Continuous variables were described by median and interquartile range. Categorical variables were described by counts and percentages, representing the count within each subgroup divided by the total number of observations (*N*) in that respective subgroup.

Statistical analysis was performed using the Mann–Whitney *U* test, the *χ*2 test, or Fisher’s exact test, depending on the data type. *P*-values < 0.05 were considered statistically significant.

Generalized estimating equations (GEE) were used for repeated measures analysis. Three distinct models were specified to identify pertinent factors. The unadjusted model solely considered the group factor (citric acid or nafamostat mesylate). Adjusted Model 1, adjusted for critical coagulation-related variables displaying significant disparities in prior analyses, was implemented. Furthermore, Adjusted Model 2 integrated significant disease severity-related factors to account for their influence.

Calculations were performed using IBM SPSS Statistics for Windows (version 28.0. Armonk, NY: IBM Corp).

## Results

### Baseline of enrolled patients

**Fig. 1:**
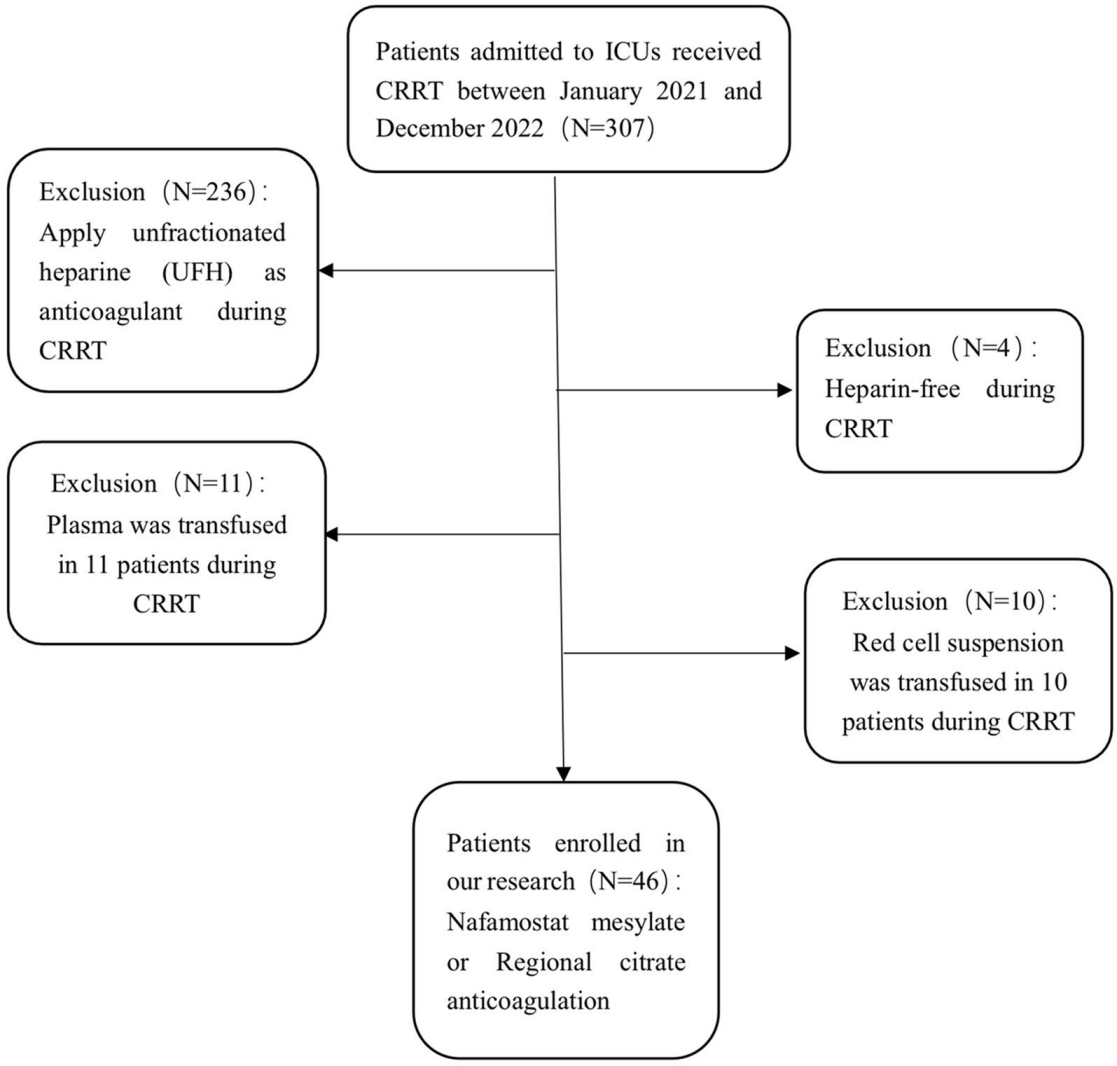
Enrollment strategy of the study

Between January 2021 and December 2022, 307 patients were screened for this study. Forty-six patients (31 men) were eventually enrolled (Fig. 1), 22 in the regional citrate group and 24 in the nafamostat mesylate group. The mean age of the patients was 65.5 (56.3, 75.3) years in the RCA group and 71.5 (63.5, 77.8) years in the NM group. Respiratory tract infection was the leading cause of sepsis in most patients. The top three causes in the regional citrate group were cerebral hemorrhage, gastrointestinal bleeding, and severe thrombocytopenia, and in the nafamostat mesylate group, gastrointestinal bleeding, severe thrombocytopenia, and coagulopathies. There were 15 patients with CKD in the regional citrate group and seven patients with CKD in the nafamostat mesylate group. Nineteen (86.4%) and 18 (75.0%) were on mechanical ventilation. Baseline characteristics were not significantly different between groups. Detailed baseline characteristics are shown in Table 1.

**Table 1:** The admission baseline characteristics of patients in both groups were comparable

Variables	Regional citrate group (*N* = 22)	Nafamostat mesylate group (*N* = 24)	*p*-value
Baseline			
Gender (male %)	15 (68.2%)	16 (66.7%)	0.913
Age (yes.)	65.5 (56.3, 75.3)	71.5 (63.5,77.8)	0.271
Height (cm)	170 (160.8, 172.5)	170 (163.0, 173.8)	0.825
Weight (kg)	65 (58.8, 80.5)	60 (50.3, 64.3)	0.063
Infection site			
Respiratory tract	9 (40.9%)	7 (29.2%)	
Urinary tract	0 (0.0%)	1 (4.2%)	
Abdominal infection	1 (4.5%)	3 (12.5%)	
Bloodstream infection	0 (0.0%)	1 (4.2%)	
Skin and soft tissue	0 (0.0%)	1 (4.2%)	
Reasons for high risk of bleeding			
Hemorrhage			
Cerebral hemorrhage	9 (40.9%)	2 (8.3%)	
Gastrointestinal hemorrhage	6 (27.3%)	5 (20.8%)	
Postoperative hemorrhage	0 (0.0%)	1 (4.2%)	
Hemoptysis	1 (4.5%)	1 (4.2%)	
Hematuresis	0 (0.0%)	1 (4.2%)	
Hemorrhoidal bleeding	1 (4.5%)	1 (4.2%)	
Hematoma	0 (0.0%)	1 (4.2%)	
Severe thrombocytopenia	3 (13.6%)	5 (20.8%)	
Coagulation disorders	2 (9.1%)	5 (20.8%)	
Postoperative surgery	0 (0.0%)	2 (8.3%)	
Underlying diseases			
Hypertension *n* (%)	14 (63.6%)	12 (50.0%)	0.351
COPD *n* (%)	2 (40%)	3 (60%)	0.711
DM *n* (%)	14 (60.9%)	9 (39.1%)	0.077
AKI *n* (%)	3 (27.3%)	8 (72.7%)	0.118
*CKD *n* (%)	15 (68.2%)	7 (31.8%)	0.008
Cancer *n* (%)	1 (4.5%)	4 (16.7%)	0.187
AF *n* (%)	0	4 (16.7%)	0.450
HF *n* (%)	5 (22.7%)	7 (29.2%)	0.619
Coronary heart disease			0.307
CAD *n* (%)	6 (27.3%)	3 (12.5%)	
ACS *n* (%)	0	1 (4.2%)	
Cerebrovascular accident			0.070
AIS *n* (%)	4 (18.2%)	1 (4.2%)	
History of CH *n* (%)	2 (9.1%)	0	
History of stroke *n* (%)	7 (31.8%)	5 (20.8%)	
Others			0.447
Acute pancreatitis *n* (%)	1 (4.5%)	0	
Liver abscess *n* (%)	2 (9.1%)	4 (16.7%)	
Critical interventions and complications			
Sepsis *n* (%)	10 (45.5%)	13 (54.2%)	0.555
Shock *n* (%)	9 (40.9%)	15 (62.5%)	0.143
MODS *n* (%)	9 (40.9%)	9 (37.5%)	0.813
Mechanic ventilation *n* (%)	19 (86.4%)	18 (75.0%)	0.332

Continuous variables were described by median and interquartile range. Categorical variables were described by counts and percentages, representing the count within each subgroup divided by the total number of observations (*N*) in that respective subgroup

Statistics analysis was conducted using the Mann–Whitney *U* test, *χ*2 test, or Fisher’s exact test. *The p-value* of < 0.05 was considered statistically significant. Calculations were performed using IBM SPSS Statistics for Windows (Version 28.0. Armonk, NY: IBM Corp)

*Yes* years, *cm* centimeters, *kg* kilograms, *COPD* Chronic Obstructive Pulmonary Disease, *DM* Diabetes Mellitus, *AKI* Acute Kidney Injury, *CKD* Chronic Kidney Disease, *AF* Atrial Fibrillation, *HF* Heart Failure, *CAD* Coronary Artery Disease, *ACS* Acute Coronary Syndrome, *AIS* acute ischemic stroke, *CH* cerebral hemorrhage, *MODS* Multiple Organ Dysfunction Syndrome

“*” means the variable was significantly different between the two groups

### Baseline laboratory indicators before CRRT of patients in the two groups

The treatment dataset consisted of measurements taken before each continuous renal replacement therapy (CRRT) session, amounting to 172 treatment applications, 97 in the regional citrate group and 75 in the nafamostat mesylate group. The HB of the patients was 74.0 g/L in the RCA group and 74.0 g/L in the NM group, the PLT of the patients was 136.0*10^9/L in the RCA group and 86.0*10^9/L in the NM group, the serum creatinine of the patients was 265. 0 umol/L in the RCA group and 138.0 umol/L in the NM group, and the APACHE II scores were 24 in the RCA and 24 in NM groups, respectively—the baseline laboratory indicators of patients in the two groups before CRRT were not significantly different. Detailed baseline characteristics are shown in Table 2.

**Table 2:** The baseline laboratory indicators before CRRT of patients in the two groups were comparable (per visit)

Variables	Regional citrate group (*N*_visit1_ = 97)	Nafamostat mesylate group (*N*_visit2_ = 75)	*p*-value
*WBC (10^9/L)	9.4 (7.3, 14.6)	12.9 (6.5, 24.4)	0.033
RBC (10^12/L)	2.5 (2.3, 2.9)	2.5 (2.2, 3.0)	0.808
HB (g/L)	74.0 (65.0, 85.0)	74.0 (64.0, 88.0)	0.581
Hct (%)	22.9 (20.1, 26.4)	22.3 (19.2, 26.9)	0.857
PLT (10^9/L)	136.0 (61.0, 216.0)	86.0 (37.0, 170.0)	0.068
*PT (S)	13.9 (12.9,15.1)	16.5 (14.9, 17.8)	0.000
*INR	1.2 (1.1, 1.3)	1.4 (1.3, 1.6)	0.000
*APTT (S)	31.6 (28.4, 37.0)	41.1 (33.7, 50.7)	0.000
Fib (g/L)	3.4 (2.3, 4.6)	3.2 (2.0, 4.8)	0.794
TT (S)	17.0 (15.9, 18.8)	17.4 (16.0, 19.5)	0.501
DD (ng/mL)	3.4 (2.2, 6.8)	4.3 (2.4, 8.9)	0.256
*Na^+^ (mmol/L)	140.0 (138.0, 145.0)	138.0 (135.0, 141.0)	0.001
K^+^ (mmol/L)	4.1 (3.8, 4.7)	4.3 (3.8, 4.8)	0.615
*BUN (mmol/L)	19.6 (11.7, 28.3)	13.3 (8.6, 21.7)	0.019
*Cr (umol/L)	265.0 (105.0, 503.2)	138.0 (96.0, 235.0)	0.000
BNP (pg/mL)	471.7 (68.6, 1292.3)	294.0 (79.2, 553.0)	0.217
*SOFA	9.0 (6.0, 12.0)	10.0 (8.0, 14.0)	0.001
APACHE II	24.0 (19.0, 27.0)	24.0 (21.5, 26.0)	0.713

The data presented in this table was derived from baseline measurements obtained before each RRT session for 46 patients distributed across two groups (Ntotal visit = 172)

Continuous variables were described by median and interquartile range

“*” means the variable was significantly different between the two groups

Statistics analysis was conducted using the Mann–Whitney *U* test. *The p-value* of < 0.05 was considered statistically significant. Calculations were performed using IBM SPSS Statistics for Windows (Version 28.0. Armonk, NY: IBM Corp)

*WBC* White Blood Cell Count, *RBC* Red Blood Cell Count, *Hb* Hemoglobin, *HCT* Hematocrit, *PLT* Platelet Count, *PT* Prothrombin Time, *INR* International Normalized Ratio, *APTT* Activated Partial Thromboplastin Time, *Fib* Fibrinogen, *TT* Thrombin Time, *DD* D-dimer, *Na* + Sodium, *K* + Potassium, *BUN* Blood Urea Nitrogen, *Cr* Creatinine, *BNP* B-type Natriuretic Peptide, *SOFA* Sequential Organ Failure Assessment, *APACHE II* Acute Physiology and Chronic Health Evaluation II, *RRT* Renal Replacement Therapy

### Anticoagulant effectiveness between two groups

The duration of CRRT was 600 min in the RCA group and 615 min in the NM group, the success rate was 90.7% in the RCA group and 85.6% in the NM group, and the difference in BUN and Cr before and after treatment between the two groups was not significantly different. Detailed data are shown in Table 3.

**Table 3:** No significant difference in anticoagulant effectiveness between two groups

Variables	Regional citrate group (*N*_visit1_ = 75)	Nafamostat mesylate group (*N*_visit2_ = 97)	*p* value
RRT duration (min)	600.0 (395.0, 716.0)	615.0 (427.5, 941.0)	0.256
Success rate (%)	90.7	85.6	0.311
△BUN (mmol/L)	3.7 (− 0.6,9.2)	1.4 (− 0.6,4.7)	0.109
△Cr (umol/L)	26.0 (− 6.7,136.0)	17.0 (− 2.0,61.6)	0.375

The data presented in this table was derived from baseline measurements obtained before each RRT session for 46 patients distributed across two groups (*N*total visit = 172). “Δ value” means the change before and after treatment. Continuous variables were described by median and interquartile range. Categorical variables were described by percentages, representing the count within each subgroup divided by the total number of observations (Nvisit) in that respective subgroup

Statistics analysis was conducted using the Mann–Whitney *U* test. *The p*-value of < 0.05 was considered statistically significant. Calculations were performed using IBM SPSS Statistics for Windows (Version 28.0. Armonk, NY: IBM Corp)

*RRT* Renal Replacement Therapy, *success rate* the rate of treatment completion rate, *BUN* Blood Urea Nitrogen, *Cr* Creatinine

### Safety of anticoagulation between two groups

There were no significant differences in the safety of anticoagulation between the two groups; for example, △Hb, △Hct, △Plt, △PT, △INR, △APTT, △TT, and △DD, there were no significant changes in hemoglobin and coagulation indices before and after treatment. Detailed characteristics are shown in Table 4.

**Table 4:** No significant difference in the safety of anticoagulation between two groups

Variables	Regional citrate group (*N*_visit1_ = 75)	Nafamostat mesylate group (*N*_visit2_ = 97)	*p* value
△Hb (g/L)	2.0 (− 3.0, 4.0)	2.0 (− 2.0, 6.0)	0.113
△HCT (%)	0.4 (− 0.8, 1.5)	0.6 (− 0.6, 2.1)	0.171
△PLT(10^9/L)	8.0 (− 2.0, 19.0)	7.0 (− 2.0, 31.5)	0.680
△PT (S)	− 0.2 (− 0.7, 0.8)	0.0 (− 1.3, 0.8)	0.589
△INR	− 0.02 (− 0.05, 0.07)	− 0.01 (− 0.10, 0.10)	0.766
△APTT (S)	− 0.7 (− 5.2, 1.3)	− 1.0 (− 4.7, 2.0)	0.896
△TT(S)	0.1 (− 1.2, 0.9)	0.0 (− 1.0, 1.0)	0.598
△DD(ng/mL)	0.01 (− 0.2, 1.2)	0.03 (− 0.4, 1.3)	0.922

The data presented in this table was derived from baseline measurements obtained before each RRT session for 46 patients distributed across two groups (*N*total visit = 172). “*Δ* value” means the change before and after treatment. Continuous variables were described by median and interquartile range. Categorical variables were described by percentages, representing the count within each subgroup divided by the total number of observations (*N*visit) in that respective subgroup

Statistics analysis was conducted using the Mann–Whitney *U* test. *The* *p-value* of < 0.05 was considered statistically significant. Calculations were performed using IBM SPSS Statistics for Windows (Version 28.0. Armonk, NY: IBM Corp)

*Hb* Hemoglobin, *HCT* Hematocrit, *PLT* Platelet Count, *PT* Prothrombin Time, *INR* International Normalized Ratio, *APTT* Activated Partial Thromboplastin Time, *TT* Thrombin Time, *DD* D-dimer

### Generalized estimating equations (GEE) analysis of the impact of different anticoagulants on the success rate of CRRT

We used Generalized Estimating Equations (GEE) to test whether different anticoagulation methods significantly affected the success rate of CRRT. In the unadjusted model, there was no statistically significant difference in the success rate of CRRT between RCA and NM, *P* > 0.05. In the Adjusted Model 1, because previous analyses showed significant differences in coagulation indices such as PT, INR, and APTT, we brought in the above variables to adjust for GEE, and the results showed no statistically significant difference in the success rate of CRRT between RCA and NM, *P* > 0.05. In the Adjusted Model 2, we further brought SOFA, WBC, and BUN, which reflect the severity of the disease, into the GEE for adjustment, and the results showed that there was no statistically significant difference between the two, *P* > 0.05 (Table 5).

**Table 5:** GEE analysis of the impact of different anticoagulants on the success rate of CRRT

	Unadjusted model			Adjusted model 1			Adjusted model 2		
Factor	B (SE)	95% wald confidence interval	*p*-value	B (SE)	95% wald confidence interval	*p*-value	B (SE)	95% wald confidence interval	*p*-value
Group (citric)	0.49	0.17–1.42	0.189	0.76	0.25–2.27	0.620	1.10	0.33–3.71	0.88
PT(S)				1.51	0.90–2.53	0.117	0.59	0.35–0.99	0.05
INR				0.02	0.00–3.43	0.130	191.67	0.86–42892.26	0.06
APTT(S)				1.03	1.01–1.06	0.007	0.97	0.94–1.00	0.36
SOFA							0.89	0.79–1.01	0.06
WBC (10^9/L)							1.06	1.00–1.13	0.04
BUN (mmol/L)							0.99	0.96–1.03	0.76

The data presented in this table was derived from baseline measurements obtained before each RRT session for 46 patients distributed across two groups (*N*total visit = 172). Different models involved different groups of factors. The Nafamostat mesylate Group was analyzed as the reference group

Statistics analysis was conducted using Generalized estimating equations (GEE) repeated measure test. *The p-value* of < 0.05 was considered statistically significant. Calculations were performed using IBM SPSS Statistics for Windows (Version 28.0. Armonk, NY: IBM Corp)

*PT* Prothrombin Time, *INR* International Normalized Ratio, *APTT* Activated Partial Thromboplastin Time, *SOFA* Sequential Organ Failure Assessment, *WBC* White Blood Cell Count, *BUN* Blood Urea Nitrogen

## Discussion

Critically ill patients may experience a variety of internal environmental disturbances. In addition to the volume, electrolyte, and acid–base imbalances often seen in AKI, severe internal environment disturbances can be caused by hepatotoxins in liver failure, pathogenic microorganisms and cytokine storms in severe infections, pathogenic antibodies in autoimmune diseases, exogenous drugs or toxins entering the body, hyperthermia in heat stroke, and so on. Using blood purification technology to restore homeostasis to maintain a good internal environment for organs and cells throughout the body will help restore organ function and improve prognosis.

In this retrospective study, nafamostat mesylate's anticoagulant efficacy and safety are not inferior to regional citrate anticoagulation. These results suggest that nafamostat mesylate can be used safely and effectively for CRRT anticoagulation in patients at high risk of bleeding.

The 2012 Kidney Disease Improving Global Outcome Clinical Practice Guidelines recommended regional citrate for AKI patients with a bleeding tendency [5]. In recent years, regional citrate, which has excellent filter life and safety performance compared with UFH, has been recommended as the first choice in Europe and the United States, even in patients with a shallow risk of bleeding [12, 13]. A small sample Randomized controlled trial (RCT) showed that regional citrate anticoagulation was safe and effective compared with an anticoagulant-free model in patients with AKI at high risk of bleeding, prolonging filter life and reducing blood loss, but further studies are needed to evaluate this [14]. An observational prospective study showed that regional citrate anticoagulation is a safe and effective method of CRRT in patients at high risk of bleeding surgery, with good cardiopulmonary bypass patency, reasonable control of acid–base status, and no clinically relevant adverse events [15]. However, using regional citrate is associated with several side effects, including hypocalcemia [12, 16, 17] and metabolic alkalosis [15]. Patients with severe liver failure [18, 19], severe hypoxemia [20], and shock with lactic acidosis are at risk of citrate accumulation [21].

Nafamostat mesylate is a trypsin inhibitor that was initially developed for the treatment of acute pancreatitis. It was later found that its protease inhibitory activity was also effective against platelet and coagulation system proteases [22–24] and is now also used for anticoagulation during cardiopulmonary bypass. Nafamostat mesylate has a low molecular weight and rapid metabolism, making it suitable for use as an anticoagulant in extracorporeal circuits during CRRT in patients at high risk of bleeding [25]. When patients at high risk of bleeding received CRRT, the anticoagulant efficacy and safety of nafamostat mesylate and non-anticoagulant mode were compared in two RCTS. It was found that nafamostat mesylate significantly prolonged the life of the extracorporeal circuit line and filter. Still, the two groups had no statistically significant difference in bleeding complications [25, 26].

Nafamostat mesylate is currently only used in China, Japan, and South Korea, and the anticoagulant effect of NM is not known in Europe or the United States. There are no retrospective observational studies or prospective randomized controlled trials comparing the anticoagulant effect of the two anticoagulants. Our ICU is one of the few units in China that can skillfully use these two anticoagulants for CRRT in patients at high risk of bleeding. In our study, the CRRT duration was 600 min in the RCA group and 615 min in the NM group, the success rate was 90.7% in the RCA group, and 85.6% in the NM group, the anticoagulant efficacy between the two groups was comparable. There was no significant difference in the safety of anticoagulation between the two groups. A common side effect of NM is hyperkalemia, which is unlikely to occur during hemodialysis due to precise solute control [27]. In this study, no patients experienced serious adverse reactions (e.g., severe anaphylaxis, eosinophilia, agranulocytosis, and myelosuppression) associated with NM administration. In addition, nafamostat mesylate has now been included in the medical insurance catalog in Shanghai, China, making it significantly cheaper than citrate acid, which must be purchased out-of-pocket.

There are several limitations to this study. First, it was a single-center study with a small sample size. Second, the analysis was retrospective, and some data baselines were different. Whether NM is superior to RCA, the results must be confirmed by a larger sample, prospective, randomized controlled trials.

## Conclusion

The anticoagulant efficacy and safety of nafamostat mesylate is not inferior to regional citrate for continuous renal replacement therapy in patients at high risk of bleeding.

## Data Availability

Data from the manuscript are not shared online. Please get in touch with correspondence authors to obtain data if necessary.

## Abbreviations

NM: Nafamostat mesylate
RCA: Regional citrate anticoagulation
CRRT: Continuous renal replacement therapy
ICU: Intensive care unit
MOF: Multiple organ failure
UFH: Unfractionated heparine
DIC: Disseminated intravascular coagulation
APTT: Activated partial thromboplastin time
KDIGO: Kidney disease: improving global outcomes
GEE: Generalized estimating equations
COPD: Chronic obstructive pulmonary disease
DM: Diabetes mellitus
AKI: Acute kidney injury
CKD: Chronic kidney disease
AF: Atrial fibrillation
HF: Heart failure
CAD: Coronary artery disease
ACS: Acute coronary syndrome
AIS: Acute ischemic stroke
CH: Cerebral hemorrhage
MODS: Multiple organ dysfunction syndrome
WBC: White blood cell count
RBC: Red blood cell count
Hb: Emoglobin
HCT: Hematocrit
PLT: Platelet count
PT: Prothrombin yime
INR: International normalized ratio
Fib: Fibrinogen
TT: Thrombin time
DD: D-dimer
Na +: Sodium
K +: Potassium
BUN: Blood urea nitrogen
Cr: Creatinine
BNP: B-type natriuretic peptide
SOFA: Sequential organ failure assessment
APACHE II: Acute physiology and chronic health evaluation II
RCT: Randomized controlled trail
